# Continuous input current buck DC/DC converter for small-size wind energy systems featuring current sensorless MPPT control

**DOI:** 10.1038/s41598-023-50692-2

**Published:** 2024-01-03

**Authors:** Nahla E. Zakzouk

**Affiliations:** Electrical and Control Engineering Department, Arab Academy for Science, Technology and Maritime Transport, Abukir, Alexandria, 1029 Egypt

**Keywords:** Energy science and technology, Engineering, Mathematics and computing

## Abstract

For decentralized electrification in remote areas, small-sized wind energy systems (WESs) are considered sustainable and affordable solution when employing an efficient, small-sized component converter integrated with a less-sophisticated, cost-effective MPPT controller. Unfortunately, using a conventional buck DC/DC converter as a MPP tracker suffer from input current discontinuity. The latter results in high ripples in the tracked rectified wind power which reduces the captured power and affects system operation especially in standalone applications which are self-sufficient and independent of grid support. Furthermore, these ripples propagate to the machine side causing vibration and torque stress which impacts turbine performance and safety. To solve this issue, a large electrolytic capacitor is placed at the buck converter input to buffer these ripples, yet at the cost of larger size, losses and reduced reliability. Oppositely, the developed C1, D4 and D6 buck converters have the merit of continuous input current at small component-size. In this paper, dynamic modelling of these three converters is developed to select the one with the least input current ripples to replace the traditional buck converter in the considered WES system. Consequently, fluctuations in the tracked power are minimized and the large buffer capacitor is eliminated. This enhances system lifetime, reduces its cost and increases tracking efficiency. Moreover, mechanical power and torque fluctuations are minimized, thus maintaining machine protection. Furthermore, a sensorless MPPT algorithm, based on converter averaged state-space model, is proposed. Being dependent on variable-step P&O algorithm, the proposed approach features simple structure, ease of control and a compromise between tracking time and accuracy besides reduced cost due to the eliminated current sensor. Simulation results verified the effectiveness of the selected converter applying the proposed MPPT approach to efficiently track the wind power under wind variations with cost-effective realization.

## Introduction

Owing to increasing energy demand, diminishing nature of fossil-fuel sources besides their environmental concerns, power generation from renewable energy sources (RESs) is gaining much interest nowadays^1^. Among various RES, wind energy, when being used to produce electric power from wind turbines, is considered one of the dispersed energy alternatives with the fastest expanding market due to its abundancy, low-cost production and minimal impact to environment^2^.

However, with its intermittent nature due to wind speed variations, capturing the most energy possible from wind energy conversion systems (WECSs) must be ensured^3^. This implies continuous tracking to the maximum power via an efficient maximum power point tracking (MPPT) algorithm realized by a reliable converter configuration^4^. To this end, a number of MPPT techniques have been developed and effectively implemented^5–11^. These methods can be classified into four main categories; direct power control (DPC), indirect power control (IPC), smart and hybrid techniques, yet each has its own advantages and limitations. Since MPPT techniques differ in many aspects (implementation complexity, accuracy, tracking speed, number of sensors, parameters dependence and prior knowledge requirement, etc.), selecting the most convenient MPPT scheme is application and system-size dependent^12^.

For standalone electrification applications in remote areas where grid access is expensive or unavailable, off-grid small-size WESs are considered one of the cost-effective solutions in locations where wind energy is abundant^12,13^. Yet, some challenging aspects should be considered to maintain high reliability, minimal complexity and reduced cost of the considered decentralized WES.

The first aspect, is the generator used in the WECS. There are various kinds of generators employed for the WES where squirrel cage induction generator (IG), doubly-fed IG and permanent magnet synchronous generator (PMSG) are the most popular ones^14,15^. Due to their high-power density, gearless operation and direct drive construction, PMSG-based WESs are an excellent candidate^16^. Moreover, their cost-effectiveness, higher reliability and efficiency, full controllability range and better fault ride-through capability make them more favorable than their counterpart^17^. Finally, being dependent on permanent magnets rather than separate excitation systems, PMSGs show more flexibility for full scale conversion.

Another challenging aspect is the applied power conversion topology. As previously discussed, wind turbines must extract the most power possible from the available wind at any given wind speed. The pitch angle of the wind turbine blade can be controlled by pitch control as one means of achieving this goal, although due to the mechanical design of small wind turbines, pitch control is somewhat problematic^18^. Therefore, achieving electrical MPPT via power conversion stages are preferable for small-scale wind turbines^19^. A passive rectifier is used with PMSG WES for low-power applications along with a DC–DC converter as a more affordable solution to control generator output power^18,19^. This DC/DC converter is responsible for the MPPT process and its performance is affected by electrical and control concerns. For a lower DC voltage level, the buck converter is a popular design especially for decentralized WECS^20^. However, typical buck converters exhibit significant limitations due to their discontinuous input current inherited feature^21^. Thus, when utilized as a MPP tracker in WES, this will result in significant tracked power ripples, thus deteriorating the MPPT process and reducing its efficiency^22^. Moreover, this is reflected on large turbine mechanical power and torque fluctuations causing harmful torque stresses which can greatly affect turbine performance and safety in standalone operation^23^. To solve this issue, a large electrolytic capacitor is placed between the rectifier output and the buck converter input to act as buffer for these fluctuations yet at the cost increasing converter size, reducing its lifetime as well as imposing electrical resonance difficulties^22^. Fortunately, in^21^, three buck DC/DC converter topologies (C1, D4 and D6) that provide continuous input continuous output (CICO) operation have been proposed. These converters would draw a regulated, ripple-free input current^24^, resulting in minimal tracked power ripples and meanwhile eliminate the need for buffer capacitor, thus improving system reliability and reducing its complexity, size and cost^25^.

Finally, employing an efficient, simple and low-cost MPPT algorithm is a further challenge facing standalone small-size WECSs which are considered in this work^26^. P&O search scheme is an appealing candidate, especially for low-power applications, since it requires only voltage and current sensors, rather than mechanical sensors, to compute changes in the tracked power and determine the perturbation direction^27^. This reduces system cost, size and implementation complexity; thus P&O is frequently deployed in commercial freestanding small-size WECSs using inexpensive microprocessors^28^. Despite its simple implementation and satisfactory performance, conventional fixed step-size P&O algorithm forces the operating point to oscillate about the MPP during rapid wind changes, which leads to high power fluctuations^27^. Hence, this limitation was addressed by replacing the constant step-size by a variable one to compromise between tracking accuracy and speed^7^.

In this paper, it is proposed to employ a continuous input current buck converter in a standalone WES, rather than the traditional buck converter, to eliminate the buffer capacitor and yet minimize tracked power ripples. To assess the three CICO buck converters (C1, D4 and D6) introduced in^21^ and select the one with least input current ripples, detailed average models are derived for each of the three converters. According to derived equations, D6 was witnessed to attain the least input current ripples i.e. highest tracking efficiency, thus will be considered in simulation work. Moreover, a current sensorless MPPT method, featuring variable-step P&O scheme, is proposed to be implemented by the selected converter to add to system simplicity and reduced cost. In summary, this paper proposes a cost-effective standalone PMSG-based WECS with the following merits;

- D6 DC/DC buck converter is applied as the MPP tracker with its continuous input current integrated capability and minimal input current ripples, thus minimizing input power ripples and maximizing tracking efficiency at the least possible component count.
- The buffer large electrolytic capacitor between the rectifier and converter stage is eliminated thus reducing system size and enhancing its lifetime and reliability.
- A current sensorless MPPT scheme is proposed which estimates the converter input current based on the state-space model of the selected D6 converter, thus reducing system size and cost
- Being dependent on variable-step P&O algorithm, the proposed MPPT scheme features the merits of simple realization, absence of any mechanical sensors and enhanced compromise between tracking time and accuracy as well as further reduction in size and cost due to the eliminated current sensor.

The proposed topology functionality was tested and validated using MATLAB/Simulink. The simulation findings confirmed that when utilized with WESs, D6 outperforms the standard buck converter; achieving minimal mechanical and electrical power oscillations while removing the large buffer capacitor. Moreover, the functionality of the proposed current sensorless MPPT controller is also verified during wind variations with a single voltage sensor rather the voltage and current sensors required by the conventional sensored controller.

## System under consideration

**Figure 1:**
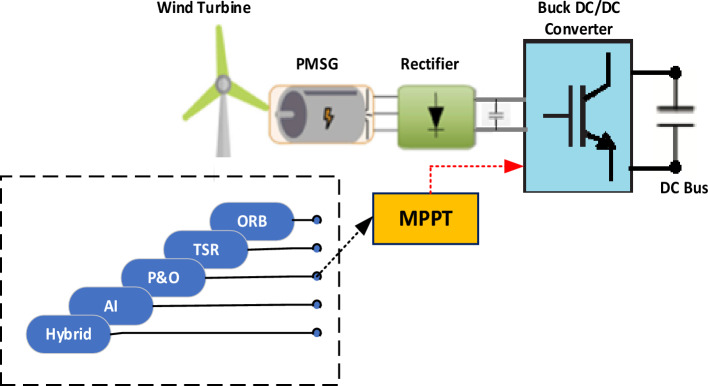
System under consideration.

Hereby, the system under consideration, shown in Fig. 1, is discussed in details^14^. It is an off-grid WECS that comprises a wind turbine, a gearless Permanent Magnet Synchronous Generator (PMSG), a passive diode rectifier and a DC/DC converter that bucks the generator rectified voltage to the required DC level and meanwhile acts as the MPP tracker. Table 1 shows considered system parameters.

**Table 1:** Considered WECS parameters.

Variable	Parameter	Value
${P}_{T}$	Rated turbine power	16 kW
${V}_{w}$	Rated wind speed	12 m/s
$R$	Turbine rotor diameter	3.14 m
${P}_{gen}$	Rated PMSG power	16 kW
${R}_{s}$	Stator resistance	0.672 Ω
${\varphi }_{f}$	Field flux linkage	2.39 wb
$J$	PMSG moment of inertia	10 kg m^2^
${f}_{c}$	PWM switching frequency	10,000 Hz
${V}_{dc}$	DC microgrid voltage	400 V
${L}_{d},{L}_{q}$	PMSG *dq* axis inductances	13.47 mH

### Wind turbine model

The mechanical power delivered by a wind turbine (WT), given ideal blades with perpendicular air flow to the rotational plane of the wind turbine, is calculated as follows^14,29^;

1 $${P}_{w}=\frac{1}{2}\rho \pi {R}^{2}{C}_{p}(\lambda ,\beta ){{v}_{w}}^{3}$$

where *ρ* is the air density in kg/m^3^, *R* is the turbine blade radius in m, *v*_*w*_ is the wind velocity striking the turbine blades in m/s and *C*_*P*_ is the turbine power coefficient.

It is worth noting that *C*_*P*_ measures the conversion efficiency of the turbine power i.e. the percentage of power that can be extracted by the WT and is limited by less than 59% as given by Betz limit^30^. As noted in Eq. (1), *C*_*P*_ depends on *λ* and *β* which are the tip speed ratio and blade pitch angle respectively. λ can be computed from Eq. (2) as follows;

2 $$\lambda = \frac{\omega R}{{v}_{w}}$$

where *ω* is the wind turbine angular mechanical speed in rad/s.

**Figure 2:**
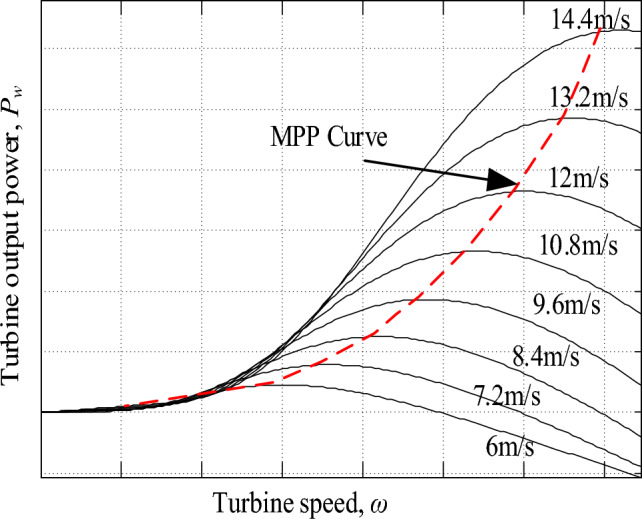
Turbine *ω*-*P* curves for different wind speeds.

Figure 2 gives the characteristic curves of harvested mechanical power from the considered WES versus WT speed for different wind speeds. It is clear the that, for each wind speed, there is an optimal power point at which the WT is forced to operate using a MPPT algorithm to extract the available peak power and maximize system efficiency.

After harvesting the maximum mechanical power, the latter is used to drive a generator to produce the required electrical energy. Due to their high-power density, high efficiency, and direct drive construction, PMSG-based WESs are an excellent candidate providing a reliable, cost-effective solution^16,17^. For successful control of generator output power in PMSG-based low-power WESs, a passive rectifier stage followed by a DC-DC converter stage is found to be a more affordable solution^18,19^.

### PMSG model

**Figure3:**
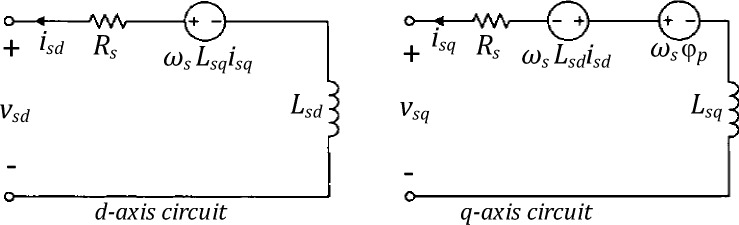
Conventional *d*–*q* coordinate frame of PMSG model: (**a**) *d*-axis circuit; (**b**) *q*-axis circuit^31^.

Considering two axis Park’s theory, the state equations governing the PMSG conventional *d*–*q* model are driven from Fig. 3 as follows^31^;

3 $$\frac{d{i}_{sd}}{dt}=-\frac{{R}_{sa}}{{L}_{sd}}{i}_{sd}+{\omega }_{s}\frac{{L}_{sq}}{{L}_{sd}}{i}_{sq}-\frac{1}{{L}_{sd}}{v}_{sd}$$

4 $$\frac{d{i}_{sq}}{dt}=-\frac{{R}_{sa}}{{L}_{sq}}{i}_{sq}-{\omega }_{s}\frac{{L}_{sd}}{{L}_{sq}}{i}_{sd}{+\omega }_{s}\frac{1}{{L}_{sq}}{\varphi }_{p}-\frac{1}{{L}_{sq}}{v}_{sq}$$

where ${{i}_{sd, }i}_{sq}$ are the *d-*axis and *q-*axis stator currents respectively, ${R}_{sa}$ is the stator resistance, ${\omega }_{s}$  is the electrical angular frequency of the generator;  ${{L}_{sd, }L}_{sq}$ are the stator inductances of generator in the *d*-axis and *q*-axis respectively; ${\varphi }_{p}$ is the permanent flux and  ${{v}_{sd, }v}_{sq}$ are the *d-*axis and *q-*axis stator voltages respectively.

The electromagnetic torque in the rotor (${T}_{e}$) is given by;

5 $${T}_{e}=1.5\frac{p}{2}\left[{\varphi }_{p}{i}_{sq}-{{i}_{sd}i}_{sq}({L}_{sd}-{L}_{sq})\right]$$

where *p* is the number of poles.

### Passive rectifier model

A full-wave bridge rectifier is applied at the generator output to convert its output AC voltage into rectified DC voltage $({V}_{r})$ which is the input voltage $({V}_{i})$ to the following buck converter stage. The rectified DC voltage is computed from Eq. (6) as follows^32^;

6 $${V}_{r}=\frac{3\sqrt{2}}{\pi }{V}_{LL}$$

where ${V}_{LL}$ is the effective value of the rectifier line-to-line input voltage.

## Conventional MPPT converter

For the considered system, a buck DC/DC converter stage is added after the rectifier stage to step-down the rectifier output DC voltage to the required DC bus level. Meanwhile the switching of this DC/DC converter stage is controlled to extract the maximum available power at the rectifier output, thus this converter is considered the MPP tracker in the considered system. Conventionally, a traditional buck converter is applied as the MPPT tracker whose switching is controlled via conventional fixed-step P&O MPPT technique.

### Conventional DC/DC buck converter model

Modeling of conventional buck converter is first carried without employing the input buffer capacitor to verify the buck integrated feature of discontinuous input current. Then, it is modelled again when applying a buffer capacitor at the buck converter input to emphasize this capacitor importance to buffer enlarged input current ripples and minimize their propagation to the machine side when using the buck converter as a MPP tracker in RES applications^33^.

**Figure 4:**
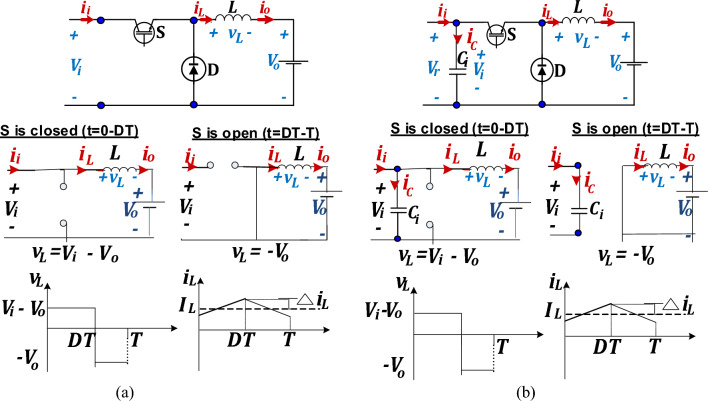
Circuit topology and switching modes for conventional buck converter (**a**) without *C*_*i*_, (**b**) with *C*_*i*_.

For each case, the converter average model is originated, in continuous inductor current mode, using Fig. 4a and b, to compute voltage and current gains, capacitor ripples voltage ripples $\Delta {{\varvec{v}}}_{{\varvec{C}}}$, inductor current ripples $\Delta {i}_{L}$ and converter input and output current ripples $\Delta {i}_{i}, \Delta {i}_{o}$ as shown below. It is worth noting that normally in the buck converter model an output capacitor is placed to filter the obtained output voltage, however, since in the considered application a DC bus or microgrid is considered, thus there is no need for an output capacitor.

- To find voltage and current transfer functions;

For both cases;

The average inductor voltage *V*_*L*_ = 0,

7 $$\left. \begin{gathered} \therefore \left( {V_{i} - V_{o} } \right)DT - V_{o} \left( {1 - D} \right)T = 0 \hfill \\ \therefore \frac{{V_{o} }}{{V_{i} }} = D \hfill \\ \end{gathered} \right\}$$

Average input power *P*_*i*_ = Average output power *P*_*o*_,

8 $$\left. \begin{gathered} \therefore V_{i} I_{i} = V_{o} I_{o} \hfill \\ \therefore \frac{{I_{i} }}{{I_{o} }} = \frac{{V_{o} }}{{V_{i} }} = D \hfill \\ \end{gathered} \right\}$$

where *V*_*i*_ and *V*_*o*_ are the converter input and output voltages respectively, *I*_*i*_ and *I*_*o*_ are the converter input and output currents respectively and *D* is the converter duty ratio.

- To find voltage and current ripples;

For Case I (without *C*_*i*_);

When the switch S is closed;

9 $$\left. \begin{gathered} v_{L} = L\frac{{\Delta i_{L} }}{DT} = V_{i} - V_{o} \hfill \\ \therefore \Delta i_{L} = \frac{{V_{o} \left( {1 - D} \right)T}}{L} = \frac{{V_{o} \left( {1 - D} \right)}}{{f_{sw} L}} \hfill \\ \end{gathered} \right\}$$

$$\Delta {i}_{o}=\Delta {i}_{L}=\frac{{V}_{o}\left(1-D\right)}{{f}_{sw}L}$$

where *f*_*sw*_ is the converter switching frequency_._

For Case II (with *C*_*i*_);

When the switch S is closed;

10 $$\left. \begin{gathered} i_{C} = C\frac{{\Delta v_{C} }}{\Delta t} = C\frac{{\Delta v_{C} }}{DT} = I_{i} - I_{o} \hfill \\ \therefore \Delta {\varvec{v}}_{{\varvec{C}}} = \frac{{I_{i} \left( {D - 1} \right)T}}{{\text{C}}} = \frac{{I_{i} \left( {D - 1} \right)}}{{f_{sw} {\text{C}}}} \hfill \\ \end{gathered} \right\}$$

11 $$\left. \begin{gathered} v_{L} = L\frac{{\Delta i_{L} }}{DT} = V_{i} - V_{o} \hfill \\ \therefore \Delta i_{L} = \frac{{V_{o} \left( {1 - D} \right)T}}{L} = \frac{{V_{o} \left( {1 - D} \right)}}{{f_{sw} L}} \hfill \\ \end{gathered} \right\}$$

12 $$\Delta {i}_{o}=\Delta {i}_{L}=\frac{{V}_{o}\left(1-D\right)}{{f}_{sw}L}=\frac{{V}_{i}D\left(1-D\right)}{{f}_{sw}L}$$

- Regarding the converter input current, it’s clear that;
  - In buck converter without input buffer capacitor *C*_i_, as shown in Fig. 4a, during the S-OFF mode, no input current exists thus the input current is discontinuous without *C*_*i*_*.*
  - In the buck converter with input buffer capacitor *C*_*i*_, as shown in Fig. 4b, during S-OFF period, the presence of buffer capacitor forced the converter input current ${i}_{i}$ to be equal to that capacitor current ${i}_{C}$ in this mode, thus overcoming its discontinuity. However, this capacitor should be relatively large to change the converter input current from discontinuous to continuous which adds to system cost and losses and affects its reliability.

### Conventional P&O MPPT method

**Figure 5:**
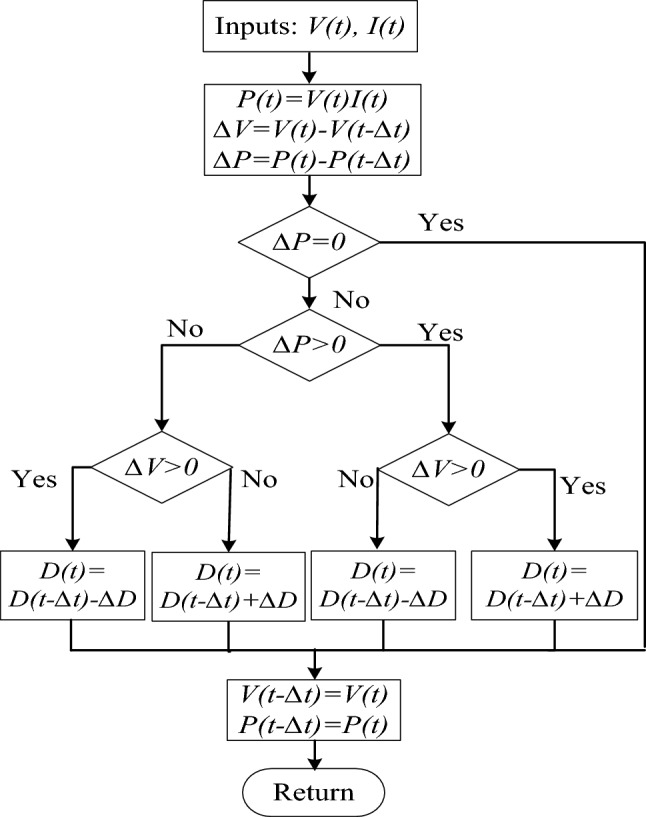
Flowchart of P&O MPPT algorithm.

Noticeably, P&O MPPT techniques are widely used in WES, especially for low-cost small-sized standalone ones, for its simplicity and flexibility, in addition to the unnecessity of distributed mechanical speed sensors or anemometers^26–28, 34^. Its tracking strategy depends on perturbing the generator output rectified voltage or current then observes the change in the extracted power to determine direction of control variable perturbation. Accordingly, the MPPT converter duty cycle will be continuously perturbed with a predetermined step size, thus regulating the DC voltage or current to maintain operation around the MPP (zero slope of the power–speed curve)^5^. Flowchart of the conventional P&O MPPT algorithm is shown in Fig. 5. However, it is worth noting that this method suffers from large power oscillations around the MPP for large step sizes and sluggish response for small ones^35^. This problem can be limited using variable step sizes which will be explained later.

## Continuous input/output power DC/DC buck converters

As previously discussed, the conventional buck converter topology suffers from input current discontinuity which results in high oscillations in the extracted power, inefficient MPPT and high torque ripples which may affect generator operation. To buffer these oscillations, a large capacitor (*C*_*i*_) is placed at the buck converter input, yet it affects the entire system life time and reliability as well as increasing its size and cost. To overcome all these limitations, the continuous input/output power DC/DC buck converters introduced in^21^ are studied and their average models are derived, in the continuous inductor current mode, to select the one with minimal input current ripples to be adopted in this study. Voltage and current gains are derived for each converter along with dynamic analysis of each converter to deduce its input and output current ripples as follows;

### D4 converter

**Figure 6:**
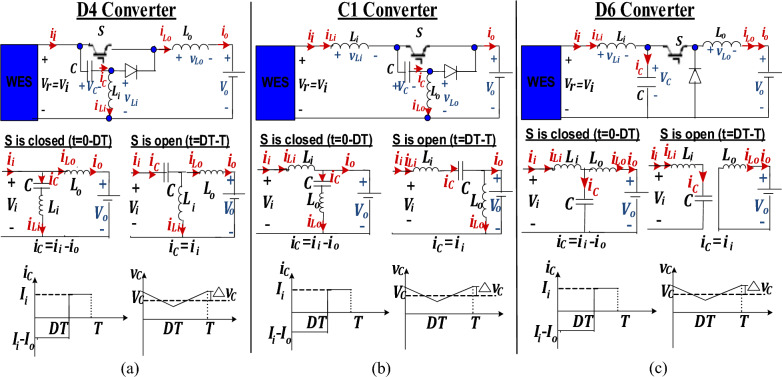
Circuit topology and switching modes for (**a**) D4, (**b**) C1, (**c**) D6 buck DC/DC converters.

Modeling of D4 converter, in the continuous inductor current mode, is originated using Fig. 6a. Voltage and current gains are computed then dynamic analysis is carried out to deduce capacitor voltage ripples $\Delta {{\varvec{v}}}_{{\varvec{C}}}$, inductors’ ripple currents $\Delta {i}_{Li}, \Delta {i}_{Lo}$ and input and output current ripples $\Delta {i}_{i}, \Delta {i}_{o}$ as shown below;

- To find voltage and current gains;

The average capacitor current *I*_*c*_ = 0,

13 $$\left. \begin{gathered} \therefore \left( {I_{i} - I_{o} } \right)DT + I_{i} \left( {1 - D} \right)T = 0 \hfill \\ \therefore \frac{{I_{i} }}{{I_{o} }} = D \hfill \\ \end{gathered} \right\}$$

Average input power *P*_*i*_ = Average output power *P*_*o*_,

14 $$\left. \begin{gathered} \therefore V_{i} I_{i} = V_{o} I_{o} \hfill \\ \therefore \frac{{V_{o} }}{{V_{i} }} = \frac{{I_{i} }}{{I_{o} }} = D \hfill \\ \end{gathered} \right\}$$

- To find the capacitor voltage ripples and inductors’ ripple currents;

When the switch S is closed;

15 $$\left. \begin{gathered} i_{C} = C\frac{{\Delta v_{C} }}{\Delta t} = C\frac{{\Delta v_{C} }}{DT} = I_{i} - I_{o} \hfill \\ \therefore \Delta {\varvec{v}}_{{\varvec{C}}} = \frac{{I_{i} \left( {D - 1} \right)T}}{{\text{C}}} = \frac{{I_{i} \left( {D - 1} \right)}}{{f_{sw} {\text{C}}}} \hfill \\ \end{gathered} \right\}$$

16 $${{v}_{Li}={L}_{i}\frac{\Delta {i}_{Li}}{\Delta t}{=L}_{i}\frac{\Delta {i}_{Li}}{DT}=V}_{i}-{v}_{C}$$

Since, average of inductor voltages = 0

17 $${\therefore v}_{C}={V}_{i} \pm \Delta {v}_{C}$$

Substitute (17) in (16);

18 $$\left. \begin{gathered} \therefore v_{Li} = L_{i} \frac{{\Delta i_{Li} }}{DT} = \mp \Delta v_{C} \hfill \\ \therefore \Delta i_{Li} = \frac{{\Delta v_{C} DT}}{{L_{i} }} = \frac{{\Delta v_{C} D}}{{f_{sw} L_{i} }} \hfill \\ \end{gathered} \right\}$$

19 $$\left. \begin{gathered} v_{Lo} = L_{o} \frac{{\Delta i_{Lo} }}{\Delta t} = L_{o} \frac{{\Delta i_{Lo} }}{DT} = V_{i} - V_{o} \hfill \\ \therefore \Delta i_{Lo} = \frac{{V_{o} \left( {1 - D} \right)T}}{{L_{o} }} = \frac{{V_{o} \left( {1 - D} \right)}}{{f_{sw} L_{o} }} \hfill \\ \end{gathered} \right\}$$

- To find input and output ripple currents;

20 $$\left. \begin{gathered} \Delta i_{i} = \Delta i_{Li} + \Delta i_{Lo} = \frac{{ \mp \Delta v_{C} D}}{{f_{sw} L_{i} }} + \frac{{V_{o} \left( {1 - D} \right)}}{{f_{sw} L_{o} }} \hfill \\ \Delta i_{o} = \Delta i_{Lo} = \frac{{V_{o} \left( {1 - D} \right)}}{{f_{sw} L_{o} }} \hfill \\ \end{gathered} \right\}$$

### C1 converter

Modeling of C1 converter, in the continuous inductor current mode, is originated using Fig. 6b. Voltage and current gains are computed then dynamic analysis is carried out to deduce capacitor voltage ripples $\Delta {{\varvec{v}}}_{{\varvec{C}}}$, inductors’ ripple currents $\Delta {i}_{Li}, \Delta {i}_{Lo}$ and input and output current ripples $\Delta {i}_{i}, \Delta {i}_{o}$ as shown below;

- To find voltage and current transfer gains;

The average capacitor current *I*_*c*_ =0,

21 $$\left. \begin{gathered} \therefore \left( {I_{i} - I_{o} } \right)DT + I_{i} \left( {1 - D} \right)T = 0 \hfill \\ \therefore \frac{{I_{i} }}{{I_{o} }} = D \hfill \\ \end{gathered} \right\}$$

Average input power *P*_*i*_ = Average output power *P*_*o*_,

22 $$\left. \begin{gathered} \therefore V_{i} I_{i} = V_{o} I_{o} \hfill \\ \therefore \frac{{V_{o} }}{{V_{i} }} = \frac{{I_{i} }}{{I_{o} }} = D \hfill \\ \end{gathered} \right\}$$

- To find the capacitor voltage ripples and inductors’ ripple currents;

When the switch S is closed;

23 $$\left. \begin{gathered} i_{C} = C\frac{{\Delta v_{C} }}{\Delta t} = C\frac{{\Delta v_{C} }}{DT} = I_{i} - I_{o} \hfill \\ \therefore \Delta {\varvec{v}}_{{\varvec{C}}} = \frac{{I_{i} \left( {D - 1} \right)T}}{{\text{C}}} = \frac{{I_{i} \left( {D - 1} \right)}}{{f_{sw} {\text{C}}}} \hfill \\ \end{gathered} \right\}$$

24 $$\left. \begin{gathered} v_{Li} = L_{i} \frac{{\Delta i_{Li} }}{\Delta t} = L_{i} \frac{{\Delta i_{Li} }}{DT} = V_{i} - V_{o} \hfill \\ \therefore \Delta i_{Li} = \frac{{V_{i} D\left( {1 - D} \right)T}}{{L_{i} }} = \frac{{V_{i} D\left( {1 - D} \right)}}{{f_{sw} L_{i} }} \hfill \\ \end{gathered} \right\}$$

25 $${v}_{Lo}={L}_{o}\frac{\Delta {i}_{Lo}}{\Delta t}{=L}_{o}\frac{\Delta {i}_{Lo}}{DT}={V}_{o}-{v}_{C}$$

Since, average of inductor voltages = 0

26 $${\therefore v}_{C}={V}_{i} \pm \Delta {v}_{C}$$

Substitute (26) in (25);

27 $$\left. \begin{gathered} \therefore v_{Lo} = L_{o} \frac{{\Delta i_{Lo} }}{DT} = V_{o} - V_{i} \mp \Delta v_{C} \mathop \Rightarrow \limits^{{\Delta v_{C} \downarrow \downarrow }} L_{o} \frac{{\Delta i_{Lo} }}{DT} = V_{o} - V_{i} \hfill \\ \therefore \Delta i_{Lo} = \frac{{V_{o} \left( {D - 1} \right)T}}{{L_{o} }} = \frac{{ - V_{o} \left( {1 - D} \right)}}{{f_{sw} L_{o} }} \hfill \\ \end{gathered} \right\}$$

- To find input and output ripple currents;

28 $$\left. \begin{gathered} \Delta i_{i} = \Delta i_{Li} = \frac{{V_{i} D\left( {1 - D} \right)}}{{f_{sw} L_{i} }} \hfill \\ \Delta i_{o} = \Delta i_{Li} - \Delta i_{Lo} = \frac{{V_{i} D\left( {1 - D} \right)}}{{f_{sw} L_{i} }} + \frac{{V_{o} \left( {1 - D} \right)}}{{f_{sw} L_{o} }} \hfill \\ \end{gathered} \right\}$$

### D6 converter

Modeling of D6 converter, in the continuous inductor current mode, is originated using Fig. 6c. Voltage and current gains are computed then dynamic analysis is carried out to deduce capacitor voltage ripples $\Delta {{\varvec{v}}}_{{\varvec{C}}}$, inductors’ ripple currents $\Delta {i}_{Li}, \Delta {i}_{Lo}$ and input and output current ripples $\Delta {i}_{i}, \Delta {i}_{o}$ as shown below;

- To find voltage and current transfer functions;

The average capacitor current *I*_*c*_ = 0,

29 $$\left. \begin{gathered} \therefore \left( {I_{i} - I_{o} } \right)DT + I_{i} \left( {1 - D} \right)T = 0 \hfill \\ \therefore \frac{{I_{i} }}{{I_{o} }} = D \hfill \\ \end{gathered} \right\}$$

Average input power *P*_*i*_ = Average output power *P*_*o*_,

30 $$\left. \begin{gathered} \therefore V_{i} I_{i} = V_{o} I_{o} \hfill \\ \therefore \frac{{V_{o} }}{{V_{i} }} = \frac{{I_{i} }}{{I_{o} }} = D \hfill \\ \end{gathered} \right\}$$

- To find the capacitor voltage ripples and inductors’ ripple currents;

When the switch S is closed;

31 $$\left. \begin{gathered} i_{C} = C\frac{{\Delta v_{C} }}{\Delta t} = C\frac{{\Delta v_{C} }}{DT} = I_{i} - I_{o} \hfill \\ \therefore \Delta {\varvec{v}}_{{\varvec{C}}} = \frac{{I_{i} \left( {D - 1} \right)T}}{{\text{C}}} = \frac{{I_{i} \left( {D - 1} \right)}}{{f_{sw} {\text{C}}}} \hfill \\ \end{gathered} \right\}$$

32 $${{v}_{Li}={L}_{i}\frac{\Delta {i}_{Li}}{\Delta t}{=L}_{i}\frac{\Delta {i}_{Li}}{DT}=V}_{i}-{v}_{C}$$

Since, average of inductor voltages = 0

33 $${\therefore v}_{C}={V}_{i} \pm \Delta {v}_{C}$$

Substitute (33) in (32)

34 $$\left. \begin{gathered} \therefore v_{Li} = L_{i} \frac{{\Delta i_{Li} }}{DT} = \mp \Delta v_{C} \hfill \\ \therefore \Delta i_{Li} = \frac{{{ }\Delta v_{C} DT}}{{L_{i} }} = \frac{{\Delta v_{C} D}}{{f_{sw} L_{i} }} \hfill \\ \end{gathered} \right\}$$

35 $${v}_{Lo}={L}_{o}\frac{\Delta {i}_{Lo}}{\Delta t}{=L}_{o}\frac{\Delta {i}_{Lo}}{DT}={v}_{C}-{V}_{o}$$

Substitute (33) in (35);

36 $$\left. \begin{gathered} v_{Lo} = L_{o} \frac{{\Delta i_{Lo} }}{DT} = V_{i} \pm \Delta v_{C} - V_{o} \mathop \Rightarrow \limits^{{\Delta v_{C} \downarrow \downarrow }} v_{Lo} \cong V_{i} - V_{o} \hfill \\ \therefore \Delta i_{Lo} = \frac{{V_{o} \left( {1 - D} \right)T}}{{L_{o} }} = \frac{{V_{o} \left( {1 - D} \right)}}{{f_{sw} L_{o} }} \hfill \\ \end{gathered} \right\}$$

- To find input and output ripple currents;

37 $$\left. \begin{gathered} \Delta i_{i} = \Delta i_{Li} = \frac{{\Delta v_{C} D}}{{f_{sw} L_{i} }} \hfill \\ \Delta i_{o} = \Delta i_{Lo} = \frac{{V_{o} \left( {1 - D} \right)}}{{f_{sw} L_{o} }} \hfill \\ \end{gathered} \right\}$$

Table 2 summarizes the considered buck converters' performance parameters. It’s vivid that the D4, C1 and D6 converters feature continuous input current unlike the conventional buck converter, thus eliminating the required buffer capacitor at converter input which in turn increases system lifetime and reduces its size and cost. However, these three converters differ regarding the input current ripples which are mirrored in the extracted power oscillations thus affecting the extracted power value and the converter tracking performance. Referring to Table 2, D6 converter experiences the least input current ripples i.e. the least tracked power oscillations. Thus, it will be selected to be applied in the considered WECS, instead of the conventional buck converter, to eliminate the need of buffer capacitor and meanwhile minimize power oscillations and maximize the tracked power.

**Table 2:** Performance parameters of considered buck converters.

	Basic buck	D4 converter	C1 converter	D6 converter
Gain	$\frac{{V}_{o}}{{V}_{i}}=\frac{{I}_{i}}{{I}_{o}}=D$	$\frac{{V}_{o}}{{V}_{i}}=\frac{{I}_{i}}{{I}_{o}}=D$	$\frac{{V}_{o}}{{V}_{i}}=\frac{{I}_{i}}{{I}_{o}}=D$	$\frac{{V}_{o}}{{V}_{i}}=\frac{{I}_{i}}{{I}_{o}}=D$
${\Delta v}_{C}$	With *C*_*i*_ ${\Delta v}_{{C}_{i}}=\frac{{I}_{i} (D-1)}{{f}_{sw}{C}_{i}}$	${\Delta v}_{C}=\frac{{I}_{i}(D-1)}{{f}_{sw}C}$	${\Delta v}_{C}=\frac{{I}_{i}(D-1)}{{f}_{sw}C}$	${\Delta v}_{C}=\frac{{I}_{i}(D-1)}{{f}_{sw}C}$
${\Delta i}_{L}$	${\Delta i}_{L}=\frac{{V}_{i}D(1-D)}{{f}_{sw}L}$	$\Delta {i}_{Li}= \frac{{\Delta v}_{C}D}{{f}_{sw}{L}_{i}}$ $\Delta {i}_{Lo}=\frac{{V}_{o}\left(1-D\right)}{{f}_{sw}{L}_{o}}$	$\Delta {i}_{Li}= \frac{{V}_{i}D\left(1-D\right)}{{f}_{sw}{L}_{i}}$ $\Delta {i}_{Lo}=\frac{{-V}_{o}\left(1-D\right)}{{f}_{sw}{L}_{o}}$	$\Delta {i}_{Li}= \frac{{\Delta v}_{C}D}{{f}_{sw}{L}_{i}}$ $\Delta {i}_{Lo}=\frac{{V}_{o}\left(1-D\right)}{{f}_{sw}{L}_{o}}$
${\Delta i}_{i}$	Without *C*_*i*_ *i*_*i*_ is discontinuous With very large *C*_*i*_*, i*_*i*_ changes from discontinuous to continuous still with large current ripples	${\Delta i}_{i}=\frac{{\Delta v}_{C}D}{{f}_{sw}{L}_{i}}+\frac{{V}_{o}(1-D)}{{f}_{sw}{L}_{o}}$	${\Delta i}_{i}=\frac{{V}_{i}D(1-D)}{{f}_{sw}{L}_{i}}$	$\Delta {i}_{i}= \frac{{\Delta v}_{C}D}{{f}_{sw}{L}_{i}}$
${\Delta i}_{o}$	${\Delta i}_{o}=\frac{{V}_{o}(1-D)}{{f}_{sw}L}$	${\Delta i}_{o}=\frac{{V}_{o}\left(1-D\right)}{{f}_{sw}{L}_{o}}$	${\Delta i}_{o}=\frac{{V}_{i}D(1-D)}{{f}_{sw}{L}_{i}}+\frac{{V}_{o}(1-D)}{{f}_{sw}{L}_{o}}$	$\Delta {i}_{Lo}=\frac{{V}_{o}\left(1-D\right)}{{f}_{sw}{L}_{o}}$

Comparing buck converter with input buffer capacitor to D6 converter, the latter shows superior performance. This can be concluded from converters’ modes of operation shown in Figs. 4b and 6c respectively and derived ripples’ equations presented in Table 2.

Regarding capacitor voltage ripples, in both cases, it depends on converter input current as deduced from Table 2. However, although a relatively large buffer capacitor is placed at buck converter input to overcome its discontinuity, still high ripples in input current exist resulting in large input voltage ripples and in turn large capacitor voltage ripples since the buffer capacitor is placed directly in parallel to converter input. Oppositely, in D6, the capacitor is separated from converter input by an input inductor which filters input current resulting in minimal capacitor voltage ripples. Hence a comparatively quite smaller linkage capacitor is required in D6 than that required at the buck converter input.

Regarding inductor current ripples, it’s clear from Table 2 that D6 input inductor current ripples, $\Delta {i}_{Li}= \frac{{\Delta v}_{C}D}{{f}_{sw}{L}_{i}}$, which is meanwhile the converter input current, just depends on the small capacitor voltage ripples, unlike the buck inductor current ripples which depend on converter input rippled voltage, ${\Delta i}_{L}=\frac{{V}_{i}D(1-D)}{{f}_{sw}L}$. Moreover, an additional output inductor exists in D6 to aid the input one resulting in low sizes for input and output inductor of D6, compared to that of buck inductor.

## Proposed MPPT converter

As previously discussed, D6 CICO power converter is applied in the considered WECS as it features minimal input current ripples. Thus, maximum tracking efficiency can be achieved without the need of buffer capacitor which reduces system size and cost and enhances system reliability. To add to these merits, D6 converter acting as the MPP tracker will use a variable-step P&O scheme to solve the tradeoff between tracking speed and accuracy. Finally, another modification is proposed to D6 control in order to eliminate the need for a current sensor by estimating the DC rectified current from D6 derived averaged state space model rather than directly measuring it. Hence, current sensorless MPPT is achieved to add to system cost effectiveness and compact size.

### D6 averaged state space model

The State Space Averaging method is widely used by the power electronics industry giving quite insight into the converter behavior^36^. The dynamic small signal model of D6 is derived based on its averaged state-space model which is divided into two sub-models, each addressing certain converter dynamics. The first sub-model analyzes the converter behavior when the converter switch (S) is at the ON state (i.e. switching period from 0: DT) while the second sub-model offers converter dynamics when the switch (S) is at OFF (i.e. switching period from DT: T). For each region, the corresponding state-space sub-model is deduced and finally the total state-space model, averaged along the total switching period, is derived. Inductors and capacitor’s internal resistances are neglected for sake of simplicity.

- *State-space sub-model when Switch “S” is ON*

From Fig. 6c, during switching ON period (t = 0-DT), the following state-space equations can be derived;

38 $${i}_{C}=C\frac{d{v}_{c}}{dt}={i}_{Li}-{i}_{Lo}$$

39 $${v}_{Lo}={L}_{o}\frac{d{i}_{Lo}}{dt}={v}_{C}-{V}_{DC-bus}$$

40 $${v}_{Li}={L}_{i}\frac{d{i}_{Li}}{dt}= {V}_{r} -{v}_{C}$$

41 $${y= {I}_{r}=i}_{Li}$$

where ${V}_{DC-bus}$ is the DC bus voltage at D6 converter output (${V}_{DC-bus}={V}_{o}$) and it is constant = 400 V while ${V}_{r}$ is the generator rectified voltage which is input to D6 converter (${V}_{r}={V}_{i}$) and has to be regulated by the MPPT controller to force operation around the MPP. Both ${V}_{DC-bus}$ and ${V}_{r}$ are considered model inputs. *y* is the model output which is the generator rectified current ${I}_{r}$ which is meanwhile D6 input current *I*_*i*_. This current is to be estimated by the sensorless MPPT controller rather than being sensed thus eliminating its sensor.

Equations (38–41) are rearranged to obtain the linear time-invariant state-space sub-model given by;

42 $$\left. \begin{gathered} \left[ {\begin{array}{*{20}c} {\dot{v}_{c} } \\ {i_{{{L}o}} } \\ {i_{{{L}i}} } \\ \end{array} } \right] = A_{1} \left[ {\begin{array}{*{20}c} {v_{C} } \\ {i_{Lo} } \\ {i_{Li} } \\ \end{array} } \right] + B_{1} \left[ {\begin{array}{*{20}c} {V_{DC - bus} } \\ {V_{r} } \\ \end{array} } \right] \hfill \\ y = C_{1} \left[ {\begin{array}{*{20}c} {v_{C} } \\ {i_{Lo} } \\ {i_{Li} } \\ \end{array} } \right] \hfill \\ \end{gathered} \right\}$$

where

$${A}_{1}=\left[\begin{array}{ccc}0& -\frac{1}{C}& \frac{1}{C}\\ \frac{1}{{L}_{o}}& 0& 0\\ -\frac{1}{{L}_{i}}& 0& 0\end{array}\right], {B}_{1}=\left[\begin{array}{cc}0& 0\\ -\frac{1}{{L}_{o}}& 0\\ 0& \frac{1}{{L}_{i}}\end{array}\right],\;and\;{C}_{1}=\left[\begin{array}{ccc}0& 0& 1\end{array}\right]$$

- *State-space sub-model when Switch “S” is OFF*

From Fig. 6c, during switching OFF period (t = DT-T), the following state-space equations can be derived;

43 $${i}_{C}=C\frac{d{v}_{c}}{dt}={i}_{Li}$$

44 $${v}_{Lo}={L}_{o}\frac{d{i}_{Lo}}{dt}=-{V}_{DC-bus}$$

45 $${v}_{Li}={L}_{i}\frac{d{i}_{Li}}{dt}= {V}_{r}-{v}_{C}$$

46 $${y= {I}_{r}= i}_{Li}$$

Equations (43–46) are rearranged to obtain the linear time-invariant state-space sub-model given by;

47 $$\left. \begin{gathered} \left[ {\begin{array}{*{20}c} {\dot{v}_{c} } \\ {i_{{{L}o}} } \\ {i_{{{L}i}} } \\ \end{array} } \right] = A_{2} \left[ {\begin{array}{*{20}c} {v_{C} } \\ {i_{Lo} } \\ {i_{Li} } \\ \end{array} } \right] + B_{2} \left[ {\begin{array}{*{20}c} {V_{DC - bus} } \\ {V_{r} } \\ \end{array} } \right] \hfill \\ y = C_{2} \left[ {\begin{array}{*{20}c} {v_{C} } \\ {i_{Lo} } \\ {i_{Li} } \\ \end{array} } \right] \hfill \\ \end{gathered} \right\}$$

where

$${A}_{2}=\left[\begin{array}{ccc}0& 0& \frac{1}{C}\\ 0& 0& 0\\ -\frac{1}{{L}_{i}}& 0& 0\end{array}\right], {B}_{2}=\left[\begin{array}{cc}0& 0\\ -\frac{1}{{L}_{o}}& 0\\ 0& \frac{1}{{L}_{i}}\end{array}\right],\;and\;{C}_{2}=\left[\begin{array}{ccc}0& 0& 1\end{array}\right]$$

- *Averaged state-space model*

For a clear insight of entire system dynamics, the total system averaged state-space model is found to be;

48 $$\left. \begin{gathered} \dot{x} = \overline{A}x + \overline{B}u \hfill \\ y = \overline{C}x \hfill \\ \end{gathered} \right\}$$

where

$$\left. {\begin{array}{*{20}c} {\overline{A} = A_{1} *d + A_{2} *\left( {1 - d} \right)} \\ {\overline{B} = B_{1} *d + B_{2} *\left( {1 - d} \right)} \\ {\overline{C} = C_{1} *d + C_{2} *\left( {1 - d} \right)} \\ \end{array} } \right\}$$

and ***d*** is the instantaneous value of D6 converter duty ratio.

Hence, applying the latter to system state space sub-models, presented in (42) and (47), the total averaged state-space model of the proposed system is given by (49);

49 $$\left. \begin{gathered} \left[ {\begin{array}{*{20}c} {\dot{v}_{c} } \\ {i_{{{L}o}} } \\ {i_{{{L}i}} } \\ \end{array} } \right] = \overline{A}\left[ {\begin{array}{*{20}c} {v_{C} } \\ {i_{Lo} } \\ {i_{Li} } \\ \end{array} } \right] + \overline{B}\left[ {\begin{array}{*{20}c} {V_{DC - bus.} } \\ {V_{r} } \\ \end{array} } \right] \hfill \\ y = \overline{C}\left[ {\begin{array}{*{20}c} {v_{C} } \\ {i_{Lo} } \\ {i_{Li} } \\ \end{array} } \right] \hfill \\ \end{gathered} \right\}$$

where

$$\overline{A }=\left[\begin{array}{ccc}0& -\frac{1}{C}d& \frac{1}{C}\\ \frac{1}{{L}_{o}}d& 0& 0\\ -\frac{1}{{L}_{i}}& 0& 0\end{array}\right], \overline{B }=\left[\begin{array}{cc}0& 0\\ -\frac{1}{{L}_{o}}& 0\\ 0& \frac{1}{{L}_{i}}\end{array}\right],\;and\; \overline{C }=\left[\begin{array}{ccc}0& 0& 1\end{array}\right]$$

**Figure 7:**
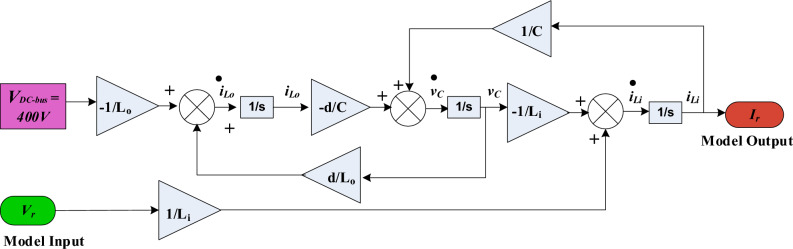
Block diagram of the derived averaged state-space model of D6 converter.

Based on the derived averaged state-space model of the selected D6 converter, the state space block diagram, shown in Fig. 7, is obtained. This diagram is the base upon which the proposed sensorless MPPT scheme is realized since the output of this model is the rectified input current of D6 converter which will be estimated using this model rather than being directly sensed using a current sensor. Conclusively, the derived averaged state-space model of D6 is used to estimate the converter input current which will be injected into the sensorless MPPT process to track the maximum power of the considered WECS. This will save the cost of the eliminated current sensor which enhances system cost-effectiveness.

### Proposed sensorless MPPT

A current sensorless MPPT scheme is proposed which consists of two main stages. In the first stage, the rectified DC current is estimated from D6 averaged state-space model derived in the previous subsection, rather than directly measuring it through a DC current sensor. Then in the second stage, the estimated rectified current along with the measured rectified voltage are utilized by a variable step P&O MPPT algorithm to extract the maximum available power and force converter operation around the MPP. The two stages are discussed in details as follows.

1. Current estimation stage

The first stage is responsible for observing and estimating the rectified generator current which is the converter input current from D6 averaged state-space-based dynamic model which is derived in the previous section. However, to achieve this, the derived model should be checked for its observability i.e. to ensure system capability of using model input and output to estimate the converter rectified current from the derived model to be injected later in the MPPT process. For the system to be completely state observable, the observability matrix Q_o_ of dimension n*n has to be full column rank i.e. its rank = *n*^37^. This means that the matrix has *n* independent pivot columns which occurs when it has a non-zero determinate i.e. $\left|{{\varvec{Q}}}_{{\varvec{o}}}\right|\ne 0$ as shown below^37^;

$$Observability\;matrix,{ }Q_{o} = \left[ {\begin{array}{*{20}c} C \\ {CA} \\ {CA^{2} } \\ : \\ : \\ {CA^{n - 1} } \\ \end{array} } \right]\mathop \Rightarrow \limits^{{\left| {{\varvec{Q}}_{{\varvec{o}}} } \right| \ne 0}} System\;is\;completely\;state\;observable$$

So, to check the observability of D6 derived model, first the observability matrix *Q*_*o*_ is computed as follows;

50 $$\left. \begin{gathered} CA = \left[ {\begin{array}{*{20}c} { - \frac{1}{{L_{i} }}} & 0 & 0 \\ \end{array} } \right] \hfill \\ CA^{2} = CAA = \left[ {\begin{array}{*{20}c} 0 & {\frac{1}{{L_{i} C}}d} & { - \frac{1}{{L_{i} C}}} \\ \end{array} } \right] \hfill \\ \therefore Q_{o} = \left[ {\begin{array}{*{20}c} 0 & 0 & 1 \\ { - \frac{1}{{L_{i} }}} & 0 & 0 \\ 0 & {\frac{1}{{L_{i} C}}d} & { - \frac{1}{{L_{i} C}}} \\ \end{array} } \right] \hfill \\ \end{gathered} \right\}$$

Hence, for an observable model from which the converter input current can be estimated $\left|{{\varvec{Q}}}_{{\varvec{o}}}\right|\ne 0 \to -\frac{d}{{L}_{i}^{2}C}\ne 0$ i.e. the duty ratio *d* $\ne 0$. Thus, the converter input current can be estimated rather than being directly sensed, eliminating the current senor.

To realize the current online estimation, the small signal model, shown in (49), is represented by the block diagram shown in Fig. 7. This block diagram presents the model in a block form rather than in matrix form thus simplifying the sensorless scheme realization. As concluded from the block diagram, the model output *I*_*r*_, which is the estimated rectified current input to D6, is estimated by measuring solely the rectified voltage *V*_*r*_ given that DC-bus voltage is constant. Thus, the current sensor can be eliminated and this control block can be implemented on a simple controller to substitute for the current sensor absence. The predicted current signal along with the measured voltage signal are fed to the controller second stage which is responsible for MPPT as described later.

**Figure 8:**
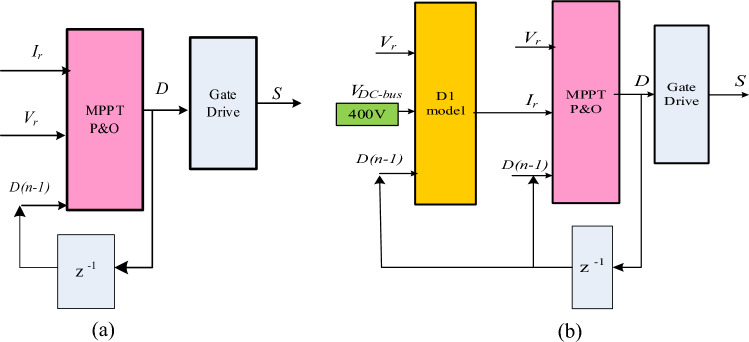
MPPT control using (**a**) Sensored MPPT controller, (**b**) Proposed sensorless MPPT controller.

Figure 8 shows the conventional sensored MPPT controller versus the proposed sensorless variable step one. Unlike the conventional single-stage MPP controller which requires both voltage and current sensors, the proposed sensorless controller requires only one voltage sensor and includes two sequential stages; current estimation stage followed by MPPT stage. Thus, the proposed current sensorless MPPT scheme has the capabilities of eliminating the current senor thus reducing the MPP controller cost and size as well as achieving noise immunity.

2. MPPT stage

The second following stage employs variable-step P&O-based MPPT algorithm to maintain the operation around the MPP. As previously discussed in the P&O scheme, the rectifier output power versus the rectified voltage is amended to operate at the zero slope for the *P–V* curve. However, for fixed step sizes^38,39^, larger step sizes result in oscillation near the MPP affecting tracking accuracy while smaller step sizes increase the tracking time and slow down the response. Thus, to solve the tradeoff between tracking accuracy and convergence speed, much research was introduced to apply steps with varying sizes according to region of operation^7^. Some use variable step sizes^40–43^, others use adaptive^44,45^ or hybrid ones^46^.

**Figure 9:**
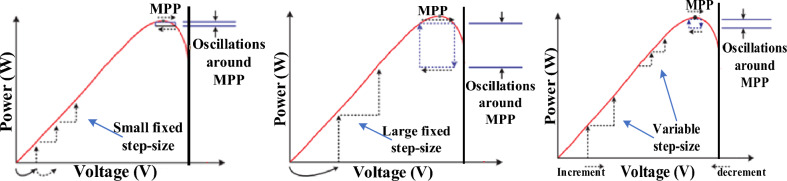
Variable-step size versus fixed step sizes for MPPT in WES.

The variable step size shown in Eq. (51) is a well-known solution to the conflict between tracking accuracy and convergence speed in PV/wind systems where both have a *P*–*V* curve that has an optimal MPP at certain environmental condition^7,22^. Meanwhile, it features easier realization than adaptive and hybrid step sizes which require more tuning and design^7^. This variable step depends on the slope of the tracked *P*–*V* curve (i.e. tracked power change divided by the tracked voltage change) which is big away from the MPP then gets smaller towards that optimal point (at which the slope of the curve = 0)^22^. Thus, larger-speed step sizes are applied in regions away from the MPP while small step sizes are utilized near the MPP as shown in Fig. 9. This eliminates the drawbacks of fixed step sizes (slow tracking for small fixed step sizes and large power oscillations around the MPP for large fixed step sizes) and enhances the WECS MPPT performance, maximizing the captured power, improving the settling time and reducing the oscillation level. Thus, for direct converter control, the conventionally adopted variable step-size $\Delta D$ is shown in (51);

51 $$\Delta D={N}_{1}\left|\frac{\Delta P}{\Delta V}\right|$$

where

$$\Delta P=P\left(k\right)-P(k-1)$$

$$\Delta V=V\left(k\right)-V(k-1)$$

$$\Delta D=D\left(k\right)-D(k-1)$$

and *N*_1_ is the scaling factor designed only once at the start of operation.

The same concept is employed but depending on the slope of the tracked *P-I* curve in WES (*dP*/*dI*)^40^ while in^42^ it depends on the slope of mechanical power of the WT versus speed (*dP*/*dω*).

However, the conventional variable step-size, being dependent on the division of PV power change by PV voltage change *(∆P*/*∆V*), can affect the MPPT performance due to this step size digression, particularly under sudden power changes due to the division issue^36^. Thus, in this paper, the variable step size is modified to depend solely on $\left|\Delta {\text{P}}\right|$ rather than $\left|\frac{\Delta {\text{P}}}{\Delta V}\right|$ as shown in (52);

52 $$\Delta D={N}_{2} \left|\Delta {\text{P}}\right|$$

**Figure 10:**
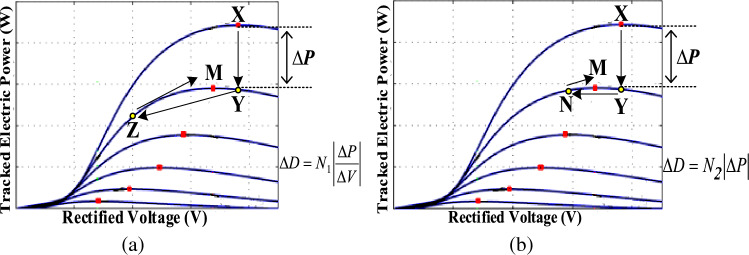
Performance of the considered MPPT algorithm when applying (**a**) *∆P/∆V* step and (**b**) *∆P* step.

This will eliminate division computations burden and also enhance the tracking performance as illustrated in Fig. 10. For a change occurring in wind speed, the operating point shifts from ‘**X**’ to ‘**Y**’. Thus, a change in rectified current (*∆I*) occurs reducing the tracked power while almost no *∆V* takes place. For a successful transfer to the new MPP ‘**M**’, the considered MPPT algorithm must decrement the duty ratio *D* by a convenient step size.

- For the widely used *∆P*/*∆V* step, an almost zero *∆V* occurs which in turn causes a vast increase in the adopted step-size. Hence, the duty ratio *D* noticeably decrements shifting operation to ‘**Z**’ which consequently results in longer tracking time till reaching ‘**M**’ and a considerable transient power loss.
- For the proposed *∆P* step, division by *∆V* is avoided, thus overcoming the large increase in the step-size. Thus, the operating point is shifted to ‘**N**’ which is close to the MPP ‘**M**’, reducing transient power loss and speeding up the tracking process.

Conclusively, the employed variable step-size minimizes tracked power ripples, enhances MPPT dynamics and simplifies control complexity due to the eliminated division computation.

Thus, the proposed sensorless MPPT controller, adopting variable-step P&O algorithm, can give satisfactory performance yet with minimum complexity for easier implementation on low-cost microcontrollers. The adopted variable step-size, given by Eq. (52), besides solving the trade-off issue, it depends solely on tracked power change *∆P* rather than the ratio *∆P*/*∆V*, thus eliminating division computations and high-power fluctuations around the MPP. Adding to all of this, elimination of current sensor adds to system compactness and cost-effectiveness.

## Simulation results

To verify the ability of selected D6 converter to minimize input current and power ripples at reduced component sizes and least conversion losses, the considered off-grid WES is simulated using MATLAB/Simulink once using the conventional buck converter (with buffer input capacitor of 220 µF and inductor of 10 mH), then again using D6 converter with reduced size passive elements (*L*_1_ = *L*_2_ = 1 mH and *C* = 10 uF). This is realized under two step changes in wind speed from 12 to 10 m/s at t = 1 s, then from 10 to 11 m/s at *t* = 2 s in order to confirm the effectiveness of the employed sensored variable-step P&O MPPT technique during sudden changes.

**Figure 11:**
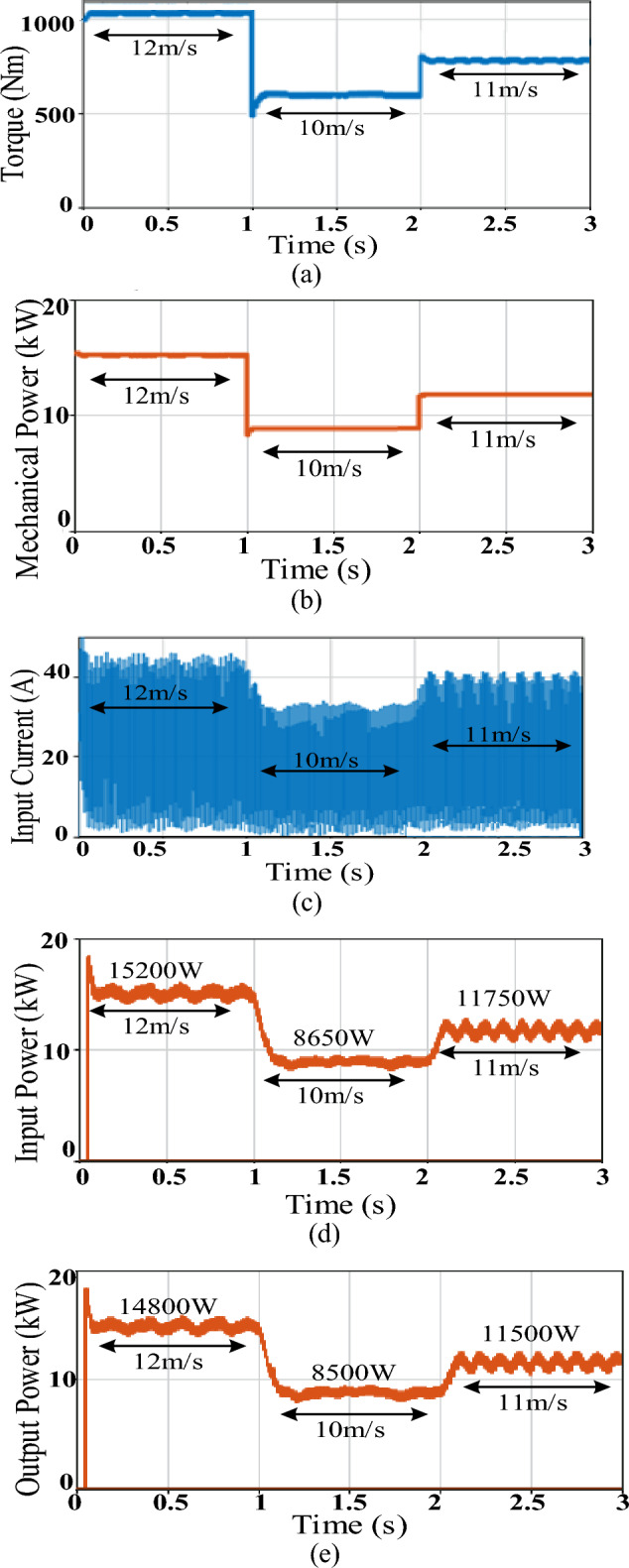
Simulation results of basic = buck converter featuring sensored MPPT; (**a**) Torque, (**b**) Mechanical power, (**c**) Converter input current current, (**d**) Converter average input power, (**e**) Converter average output power.

**Figure 12:**
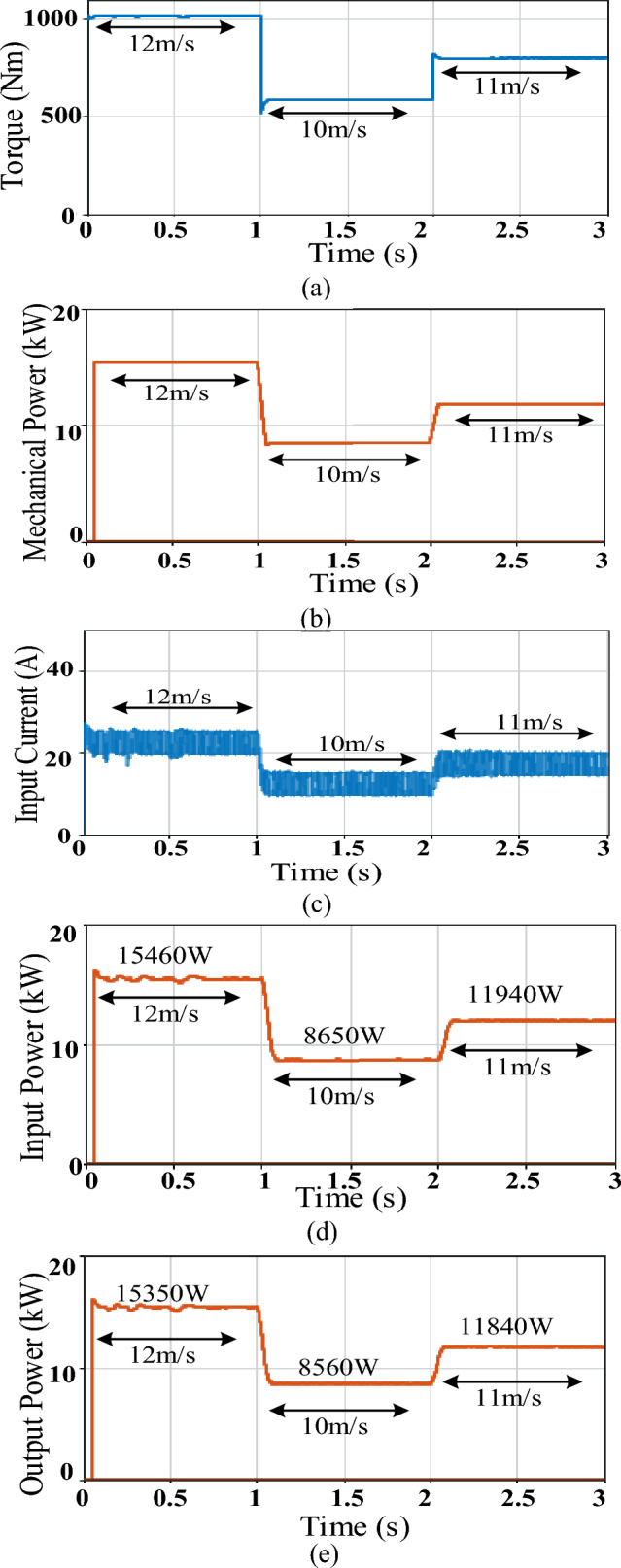
Simulation results of D6 converter featuring sensored MPPT; (**a**) Torque, (**b**) Mechanical power, (**c**) Converter input current, (**d**) Converter average input power, (**e**) Converter average output power.

Figure 11 shows system simulation results using the basic buck converter while Fig. 12 demonstrates those of D6 converter. Simulation results include generator torque and mechanical power, converter input current, converter average input tracked power and converter average output power. Table 3 summarizes simulation results’ parameters that include the attained torque ripples $\Delta {\varvec{T}}$, mechanical power ripples $\Delta {P}_{mech}$, converter input current ripples $\Delta {I}_{i}$, converter average input power and its ripples ${P}_{i},\Delta {P}_{i}$ and converter average output power and its ripples ${P}_{o},\Delta {P}_{o}$ for each of the two converters. The converter average input power ${P}_{i}$ shows the power that was successfully tracked by the converter and available at its input where its value is enhanced by the converter ability to minimize the input current ripples. Finally, the converter efficiency which is affected by the converter switching and copper losses is computed by (53). The less the inductor size, the less their copper losses, thus the more available power at the converter output ${P}_{o}$.

**Table 3:** Components values and performance parameters for considered converters during simulation tests.

	Buck (sensored MPPT)			D6 (sensored MPPT)			D6 (sensorless MPPT)		
*L*	*L* = 10 mH *R*_*L*_=0.1Ω			*L*_1_ = *L*_2_ = 1 mH *R*_*L*1_=* R*_*L*2_ = 0.01 Ω			*L*_1_ = *L*_2_ = 1 mH *R*_*L*1_ = *R*_*L*2_ = 0.01 Ω		
*C*	*Ci* = 220 μF			*C* = 10 μF			*C* = 10 μF		
Settling time for 1st change	0.2 s			0.06 s			0.15 s		
Settling time for 2nd change	0.1 s			0.07 s			0.08 s		

Performance parameters at different wind speeds	12 m/s	10 m/s	11 m/s	12 m/s	10 m/s	11 m/s	12 m/s	10 m/s	11 m/s
$\Delta {T}_{mech}$ (Nm)	± 7	± 5	± 7	± 0.5	± 0.75	± 0.6	± 2	± 1	± 0.4
$\Delta {P}_{mech}$ (W)	± 50	± 12	± 50	± 1.5	± 5	± 3	± 12	± 10	± 8
$\Delta {I}_{i}$ (A)	± 15	± 12.5	± 15	± 3	± 2.8	± 2.5	±4.5	± 3	± 2.5
${P}_{i}$ (W) $\Delta {P}_{i}$ (W)	15200 ± 500	8650 ± 300	11750 ± 600	15460 ± 20	8650 ± 50	11940 ± 35	15400 ± 100	8550 ± 80	11800 ± 50
${P}_{o}$ (W) $\Delta {P}_{o}$ (W)	14800 ± 500	8500 ± 300	11500 ± 600	15350 ± 20	8560 ± 50	11840 ± 35	15300 ± 120	8500 ± 75	11760 ± 50
${\mathrm{\% \xi }}_{{\text{conversion}}}$	97.4%	98.3%	97.9%	99.3%	99%	99.2%	99.35%	99.4%	99.67%

53 $${\mathrm{\%}\xi }_{conversion}=\frac{{P}_{o}}{{P}_{i}}\left(100\right)$$

Analyzing Figs. 11 and 12 parts along with Table 3 parameters, both converters were able to adequately track the MPP, during different wind speeds, which validates the applied sensored variable-step P&O scheme. However, there were differences in performance between both converters as explained below.

First regarding the inductor and capacitor sizes, the ones included in the basic buck converter have relatively larger values than those in D6, thus more cost, size and losses. This was selected to reduce the effect of the input current discontinuity of the basic buck converter on its MPPT performance, yet at the cost of more losses and reduced lifetime. Moreover, the basic converter shows relatively slower tracking time than that of D6 due to its larger passive elements which increase the time constant and in turn slows down the response as concluded from Table 3 regarding the less settling time attained by D6 during both step changes.

Despite its larger size passive elements, still noticeably large fluctuations in the buck converter input current exist as shown in Fig. 11c compared to those of D6 input current given by Fig. 12c. These ripples are reflected in the relatively large fluctuations in the generator torque and mechanical power of the basic buck converter (Fig. 11a and b respectively) when compared to those of D6 (Fig. 12a and b respectively), as presented in Table 3, which in turn affects turbine safety. On the other hand, the less fluctuations in D6 converter reduces stresses on the machine and maintains its lifetime.

Moreover, the larger input current ripples in basic buck converter are also mirrored in considerably more power ripples at the converter input thus reducing the available tracked input power (Fig. 11d) when compared to the available input power of D6 with its minimal oscillations (Fig. 12d). This enhanced the tracking performance of D6 during different wind speeds.

Finally, the relatively less size passive elements included in D6 feature less losses i.e. more power available at the converter output during different wind speeds (Fig. 12e when compared to that of basic buck (Fig. 11e). Thus, as shown in Table 3, D6 exceled in the conversion efficiency achieved, experiencing ≥ 99% at different wind speeds. Moreover, remarkable increase in D6 output power can be noticed when compared to that of the traditional buck converter i.e. D6 output power exceeds that of basic buck converter by almost 550, 340, and 60 W at wind speed of 12, 11 and 10 m/s respectively.

These enhancements are related to D6 converter outstanding property of continuous input current which is further reflected on minimal ripples in the converter input power and more available power to be tracked in addition to eliminating the need of the buffer large capacitor. Thus, the whole system reliability, efficiency and tracking time are enhanced along with reducing stresses on machine.

Another experiment is carried out to verify the effectiveness of the proposed sensorless variable-step P&O MPPT scheme. In this experiment, the superior D6 converter is again tested using MATLAB/Simulink under the two considered step changes of wind speed but when applying the proposed sensorless MPPT scheme based on its derived state space averaged model of D6 converter.

**Figure 13:**
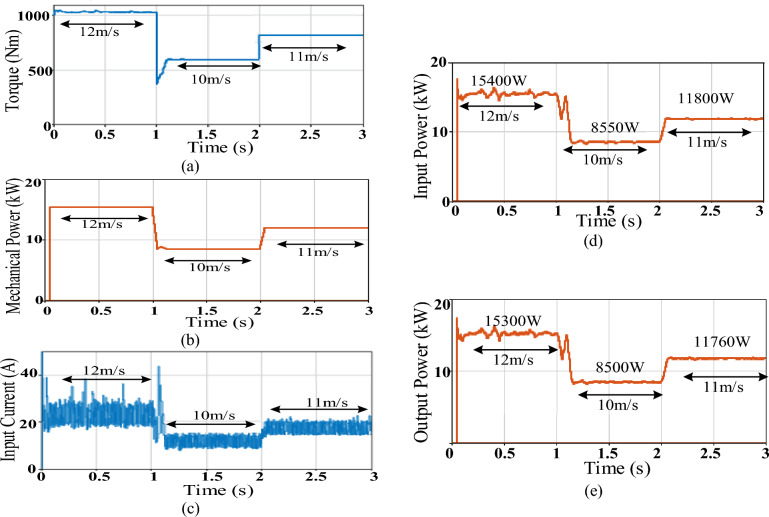
Simulation results of D6 converter featuring sensorless MPPT; (**a**) Torque, (**b**) Mechanical power, (**c**) Converter input current, (**d**) Converter average input power, (**e**) Converter average output power.

Figure 13a–e shows D6 simulation results, when applying the proposed sensorless MPPT scheme, regarding generator torque and mechanical power, converter input current, converter average input tracked power and converter average output power respectively while the third column of Table 3 summarizes D6 performance parameters for these simulation results.

It can be concluded that using the proposed sensolress MPPT scheme, D6 successfully tracked the MPP during different wind speeds which validates the proposed scheme principle. However, longer tracking time and more ripples are noticed with D6 when using the sensorless controller rather than the sensored one. This can be related to the computations burden and time required to estimate the converter input current value by the sensorless scheme. Despite the small differences between both controllers’ response, the overall performance of both is quite close verifying the proposed sensorless one effectiveness due to its less cost and size. Moreover, D6 converter applying the sensorless scheme still outweighs the basic converter regarding the tracking time, the ripples attained in the applied torque, mechanical power and input current as well as the input and output converter powers’ values. It was able to achieve conversion efficiencies of more than 99% during different wind speeds.

In summary, simulation results demonstrated how the selected CICO D6 converter outweighs the conventional buck one regarding current continuity, minimal electrical and mechanical power ripples and enhanced system reliability and efficiency yet at considerably less capacitor and inductor values. Moreover, the effectiveness of proposed sensorless controller is verified in tracking the MPP during different wind speeds with quite acceptable performance parameters adding to system compactness and cost effectiveness due to the eliminated current sensor.

## Discussion

Generally, small sized decentralized wind energy-based power generation systems require efficient and reliable MPP tracker realized with simple implementation and low-cost microcontrollers. Thus, in this paper, the considered standalone PMSG-based WECS adopts a continuous input current buck converter featuring minimal input current ripples without the need input buffer capacitor, thus system reliability and tracking efficiency are enhanced. Moreover, a current sensorless MPPT controller, based on variable step P&O algorithm, is proposed to add to system simplicity and reduced cost as well as compromise between the tracking time and accuracy. The proposed system capabilities can be summarized as follows;

- Selected D6 converter

Among continuous input current buck converters, D6 converter was selected since it features the least input current ripples, as verified from the derived average models of these converters. This results in least fluctuations in the tracked power and better tracking performance. Compared to traditional two-passive elements buck converter, the selected three-element D6 converter, despite its greater component-count, features less cost, losses and enhanced reliability as well as better MPPT performance as demonstrated below.

According to Table 4, the values of passive elements selected in simulation work for the buck converter and D6 are listed. It’s clear that the input capacitor and output inductor of the conventional buck converter outsize those of D6 converter (the converter capacitor and input and output inductors). Despite these relatively larger values, more input current and power ripples have resulted in case of the basic buck converter as verified by simulation results. However, if larger values for buck converter elements are to be selected, system cost and size will greatly increase and the system will be cost-ineffective. Thus, values shown in Table 4 are adopted.

**Table 4:** Components’ sizes selected for simulation work for buck and D6 converters.

Buck	D6
*L* = 10 mH, *R*_*L*_ = 0.1 Ω	*L*_1_ = *L*_2_ = 1 mH *R*_*L*1_ = *R*_*L*2_ = 0.01 Ω
*C*_*i*_ = 220 μF	*C* = 10 μF

Although there may be some variations in selected elements’ sizes in real-time implementation, yet still buck converter will possess elements of larger size than those of D6. So, the objective of this comparison is to show the effect of element size on element cost and losses and that although D6 is a three-passive element converter, it is less in cost and losses than the two-element buck since it acquires less elements size. For fair comparison, same operating conditions are considered as well as same components’ manufacturer in both converters w.r.t to each element type (capacitor or inductor).

i. *Cost*
  Regarding the cost issue, it varies according to different aspects, mainly component size and operating voltage/current level. According to values presented it Table 4, the cost of each converter passive component is decided based on the prices shown on the website of the trust-worthy electric components’ store; “Mouser electronic components”, Available at: https://eu.mouser.com/.
  Capacitors’ cost
    The input capacitor of the buck converter should withstand operating voltage of almost 620 V which is the rectified voltage *V*_*r*_, at the converter input (*P*_*r*_/*I*_*r*_ = 15500 W/25 = 620 V). Thus, a 220 µF, 620 V capacitor is required. For cost-effectiveness, an electrolytic capacitor with close specifications (220 uF, 630 V) was found for almost 8€ as shown in Table 5. So, it was selected, yet at the limitation of more size and weight as well as less lifetime and reliability than a corresponding film one due to high input current ripples at the converter input where it is placed. For better reliability, the electrolytic capacitor can be replaced by a film capacitor featuring close specifications of 225 uF, 700 V yet at a higher cost of 85.74€ which is more than ten times that of the electrolytic one as shown in Table 5.

**Table 5:** Specifications of capacitors employed in buck and D6 converters.

Converter	Capacitor	Part number	ESR	Cost	Manufacturer	Photo
Buck	220 µF, 630 V Aluminum Electrolytic Capacitor	ALF70C221DE630	596 mΩ	8.01 €	KEMET	
	225 µF, 700 V Polypropylene (PP) film capacitor	C44UJGT6225A8TK	1.2m Ω	85.74 €	KEMET	
D6	10 µF, 700 V Polypropylene (PP) film capacitor	C4AQJBU5100M11J	6.8m Ω	4.15 €	KEMET
    Regarding D6 converter with same operating conditions only 10 µF is required as per Table 4. Thus, a film capacitor of close specifications (220 µF, 630 V) is chosen for its reliability and relatively much less cost of 4,15€ which is half the cost of buck electrolytic converter and twenty times less than buck film capacitor cost as shown in Table 5. This is related to the smaller size of D6 capacitor compared to that of buck.
  Inductors’ cost
    The conventional buck output inductor should withstand operating current of almost 40 A which is the converter output current (*P*/V_o_ = 15500 W/400 = 39 A). Thus, according to Table 4 values, a 10 mH, 40 A inductor is required for buck converter whereas for D6, a 1 mH, 40 A output inductor and 1 mH, 25 A input inductor are required. However, according to components availability, the following was found at Mouser website;
    For buck converter, a 50 A, 10 mH, 0.023 Ω power inductor is available for 760€ whereas for D6 input inductor, a 30 A, 1 mH, 0.009 Ω is available for 96€ and a 50 A, 1 mH, 0.005 Ω for 163.6€ is available for D6 output inductor as shown in Table 6. Hence, it’s clear that the cost of D6 both inductors’ (260€) is still almost three times less than the cost of the single buck inductor due to its larger size. Moreover, the 10mH buck inductor features resistance of 0.023 Ω which is almost five times that of D6 output inductor and 3.5 times that of D6 input inductor as concluded from Table 6 which results in more losses in buck converter.

**Table 6:** Specifications of inductors employed in buck and D6 converters.

Inductor type	Converter	*L*	Part number	*R*	Cost	Manufacturer	Photo
Power Inductor	Buck	10 mH, 50 A power inductor	195J50	0.023 Ω	760€	Hammond Manufacturing	
	D6	*L*_*i*_ 1 mH, 30 A power inductor	195C30	0.009 Ω	96€	Hammond Manufacturing	
		*L*_*o*_ 1 mH, 50 A power	195C50	0.005 Ω	163.6€	Hammond Manufacturing	
Choke	Buck	10 units of 1 mH, 50 A Common Mode Choke	RT8122-50-1M0	10* 1.7 mΩ	10* 11.45€	Schaffner	
	D6	*L*_*i*_ 1 mH, 50 A Common Mode Choke	RT8122-50-1M0	1.7 mΩ	11.45€	Schaffner	
		Lo 1 mH, 50 A Common Mode Choke	RT8122-50-1M0	1.7 mΩ	11.45€	Schaffner
    To reduce the inductor cost, a 50 A, 1 mH, 0.0017 Ω common mode choke inductor is available at 11.45€, yet at the size of 1mH and not available in bigger sizes. Hence, it can be used for the input and output inductors of D6 with total cost of 2*11.45€. However, when applied with the buck, it can be used as a series patch of 10 inductor units to realize total of 50 A, 10*1 mH, 10*0.0017 Ω inductor yet at a bigger size and weight as well as five times the cost of that of D6 in addition to more resistive losses.
    Conclusively, the larger capacitor and inductor sizes featured by the two-element buck converter cost way more than those of the three-element D6 due to the latter relatively much smaller sizes.
ii. *Losses*
  As per resistive power losses associated with converters’ capacitors, almost close ESR resistances are experienced in the film capacitor associated with buck or D6, yet the high voltage ripples buffered by buck capacitor can increase its losses. However, for cost-effectiveness, if an electrolytic capacitor is used with buck, it features a quite bigger ESR resistance as shown in Table 5 i.e. more losses. Normally, electrolytic capacitors have larger internal power loss for same amount of ripple current compared to film ones^47^.
  Regarding resistive power losses experienced by converters’ power inductors, the input and output power inductors of D6 exert relatively less resistances values (*R*_*Li*_ = 0.009 Ω, *R*_*Lo*_ = 0.005 Ω) compared to that of buck output inductor (*R*_*L*_ = 0.023 Ω), as shown in Table 6, resulting in total average power losses for both D6 inductors altogether less than that exerted by the buck single inductor.
  For *I*_*i*_ = 25 A and *I*_*o*_ = 40 A,
  $$For\;D6;P_{loss - D6} = I_{i}^{2} \left( {0.009} \right) + I_{o}^{2} \left( {0.005} \right) = 13.6\;{\text{W}}\;whereas\;for\;buck\;P_{loss - buck} = I_{o}^{2} \left( {0.023} \right) = 36.8\;{\text{W}}$$
  As per losses experienced by converters’’ choke inductors, the average losses exerted by buck converter inductor units are quite larger than those of D6 input and output inductors altogether since buck is made of 10 series inductor units as shown in Table 6 while each inductor in D6 is a single unit (as in simulation case).
  For *I*_*i*_ = 25 A and *I*_*o*_ = 40 A,
  $$For\;D6;P_{loss - D6} = I_{i}^{2} \left( {0.0017} \right) + I_{o}^{2} \left( {0.0017} \right) = 3.78\;{\text{W}}\;whereas\;for\;buck\;P_{loss - buck} = 10I_{o}^{2} \left( {0.0017} \right) = 27.2\;{\text{W}}$$
  Conclusively, although buck has two passive elements rather than the three-element D6, it acquires more resistive power losses due to its components’ relatively larger size.
iii. *Reliability*
  As previously explained in the cost aspect, for cost effectiveness, the buck film capacitor can be replaced by electrolytic capacitor. However, electrolytic capacitors experience less lifetime affecting reliability in case of high current ripples which is the case of the buck converter^47^. The application of excessive mechanical stress or excessive electrical parameters such as operating voltage and ripple currents cause poor contact or open circuits in electrolytic capacitors causing its degradation & shortens its life span^48^.
iv. *MPPT performance*
  Although the buck converter features larger passive elements than those of D6, still it experiences larger input current ripples as shown in Fig. 11c compared to that of D6, Fig. 12c due to buck converter inherited feature of pulsating input current. This results in larger ripples in the power tracked by buck converter, more losses and less captured power which affects system efficiency as concluded from simulation results summarized in Table 3. Moreover, these fluctuations are mirrored in relatively larger mechanical power and torque fluctuations which affect turbine safety.

In summary, despite having less component-count, the conventional buck experiences more cost, losses and less reliability than D6 due to the former relatively larger components’ sizes. Moreover, despite larger passive elements employed by the traditional buck converter, it still experiences larger power oscillations and torque fluctuations. Thus, D6 has merits of reduced cost, size and losses, enhanced reliability, efficiency and tracking performance as well as less stress on machine which are appealing features for off-grid self-sufficient RES applications where power supply is totally independent of utility.

- Proposed current sensorless P&O-based MPPT scheme
  P&O MPPT algorithm is very popular in small-size, low-cost systems since, in such systems, it gives successful tracking performance with the merits of simple implementation and control, thus can implemented using low-cost microcontrollers. Moreover, no mechanical sensors or prior knowledge of WT parameters are required since it depends solely on measuring the rectified voltage and current to achieve accurate MPPT. Thus, in this study, P&O is adopted. However, an added merit is proposed to the applied scheme where the current sensor is also removed and the converter input current is estimated using the state-space model of the selected D6 converter, Thus, further reduction to system size and cost is realized without affecting the MPPT process as verified by simulation results.
- Variable step-size
  Perturbing the control variable in the MPPT process, which is the converter duty ratio, using the proposed variable step size solves the trade-off between extracted power oscillations and the tracking convergence speed. Moreover, eliminating the division in the step size reduces the computational burden thus decreasing software complexity. A number of studies applied P&O based MPPT schemes in WECS^38–46^. Fixed step-sizes^38,39^ result in either high power oscillations around the MPP for fast tracking or low convergence speed for less ripples. Thus, selecting the step-size is challenging and tracking performance is greatly affected. To solve this issue, the fixed step-size is replaced by variable^40–43^, adaptive^44,45^ or hybrid ones^46^.
  Adaptive step sizes are defined for each perturbation depending on a definite objective function which clarifies the relation among control variables and wind speed. This function can be dependent on multiple constants which need to be accurately tuned, thus increasing control complexity. Hybrid step sizes result from the combination of step-sizes of different types while MPP tracking is employed. However, their operation order and activation need accurate design which again adds to control complexity. Although adaptive and hybrid step sizes achieve very good compromise between oscillations and speed, their tuning and design requirements add to implementation complexity and require user experiene^7^. Featuring less complexity, variable step-sizes, are simpler in realization than the latter and their tuning requirements are minimal^7^. Meanwhile low oscillations around the MPP and high tracking speed are well addressed. Hence, in the proposed MPPT scheme, variable step-size is selected since it combines between less complexity, minimal design requirements and successfully solves the tradeoff issue.
  Table 7 compares different P&O schemes applied to WECS, presented in literature, with the proposed one. The proposed current sensorless MPPT scheme, based on division-free variable step-size P&O algorithm, combines between simple implementation, efficient tracking performance and a good compromise between tracking speed and power oscillations yet at the minimal design requirements and component count. Notably, due to the eliminated current sensor, it outweighs the others regarding size and cost. However, it is worth noting that the proposed sensorless scheme is converter dependent since it depends on the applied converter state space model i.e. changing the converter topology implies to derive the new converter state space model to help estimate the rectified current rather than measuring it.

**Table 7:** Comparison of recent P&O MPPT schemes, introduced in literature for WESC, with the proposed one.

Work	Adopted step size	Complexity	Solves the trade-off issue	Tuning requirements	V and I sensors
^38,39^	Fixed	Low	No	Challenging	*V*, *I*
^44,45^	Adaptive	High	Yes	High	*V*, *I*
^46^	Hybrid	High	Yes	High	*V*, *I*
^40–43^	Variable	Moderate	Yes	Low	*V*, *I*
Proposed	Variable	Moderate	Yes	Low	*V* only

## Conclusions

For decentralized WECS, the implemented converter topology, its input power continuity and the employed MPPT algorithm have a significant impact on system electric power tracking performance, conversion efficiency as well as its produced mechanical power and torque nature. Thus, in this paper, three continuous input/continuous output power buck converters (D4, C1 and D6) are studied and their dynamic models are derived to select the one with the least input current ripples. D6 converter was selected since it shows minimal input power ripples and in turn best tracking performance. Moreover, a current sensorless MPPT algorithm is proposed to be applied on the selected converter topology. Being dependent on division-free variable-step P&O algorithm, the proposed MPPT scheme features the merits of simple realization, absence of any mechanical sensors and enhanced compromise between tracking time and accuracy as well as reduced size and cost due to the eliminated current sensor. Simulation results verify the superiority of the selected D6 converter when compared with the traditional buck achieving minimal mechanical and electrical power oscillations with less inductor and capacitor sizes, all of which enhance tracking time and system reliability as well as increasing the available tracked power and decreases system total cost and losses. Finally, the functionality of the proposed current sensorless MPPT controller is also verified using simulation results during varying wind speeds which adds to system cost effectiveness and compactness.
